# Benefits of surgical treatment within 48 h of proximal femoral fracture in centenarians: a retrospective cohort study

**DOI:** 10.3389/fsurg.2024.1349434

**Published:** 2024-02-27

**Authors:** Toshiya Shitahodo, Shizumasa Murata, Yoji Kitano, Yoshimasa Mera, Hiroki Iwahashi, Shingo Inoue, Kota Kawamura, Hiroshi Yamada

**Affiliations:** ^1^ Department of Orthopedic Surgery, Shingu Municipal Medical Center, Shingu, Wakayama, Japan; ^2^ Department of Orthopedic Surgery, Wakayama Medical University, Wakayama, Japan

**Keywords:** proximal femoral fractures, centenarians, walking ability, postoperative life expectancy, osteoporosis

## Abstract

**Introduction:**

Proximal femoral fractures in aging populations represent a significant concern, with an increasing prevalence among individuals aged ≥100 years. The existing research does not provide robust guidance for clinicians managing older patients aged ≥100 years with proximal femoral fractures. We investigated the safety and efficacy of surgical treatment in patients aged ≥100 years with proximal femoral fractures and evaluated the impact of early surgery on their outcomes.

**Methods:**

This retrospective cohort study involved 15 patients aged ≥100 years who underwent surgical treatment of proximal femoral fractures; the control group included 137 patients in their 90s. Data were collected between January 2010 and December 2017. Evaluation items included patient characteristics, surgical details, perioperative complication rates, length of hospital stay, the proportion of patients discharged to the same facility or home, rate of regaining walking ability, and 1-year survival rate.

**Results:**

The patients aged ≥100 years and those in their 90s had comparable outcomes. Thus, age alone does not dictate surgical success. Early surgery (≤48 h) was associated with trends toward improved perioperative complications, ambulatory ability, and return to original living environment.

**Discussion:**

This study underscores the potential benefits of surgical intervention for proximal femoral fractures in patients aged ≥100 years, indicating the relevance of early surgery (≤48 h). Our findings emphasized the importance of timely intervention and evidence-based decision-making for this demographic. Clinicians, policymakers, and patients could benefit from our insights to enhance fracture management strategies, along with future research endeavors to validate and expand our results in larger multicenter cohorts.

## 1 Introduction

The incidences of proximal femoral fractures and number of patients with these fractures, a debilitating injury among older adults, have markedly increased with an aging population (1). Cases among individuals ≥100 years of age are no longer rare, highlighting the immense challenges facing a super-aged society (2). Although a considerable body of literature exists on the surgical treatment of proximal femoral fractures in older adults, there remains a notable need for more evidence specifically pertaining to older patients aged ≥100 years (3). Consequently, the specific impact of surgical intervention on postoperative life expectancy, changes in walking ability, and alterations in living environments in this age group remains largely uncertain. Additionally, although early surgery within 48 h is reported to offer numerous benefits to patients with proximal femoral fractures, there are only a few reports on the efficacy of early surgery in patients aged ≥100 years (4, 5).

The existing literature has provided valuable insights into surgical interventions for proximal femoral fractures in older adults, clarifying mortality rates, functional recovery, and healthcare resource utilization within this population (6, 7). These studies have underscored the significance of comprehensive preoperative assessment, appropriate surgical approaches, and postoperative rehabilitation strategies for optimizing outcomes (8, 9). However, the available evidence fails to provide robust guidance for clinicians managing older patients aged ≥100 years with proximal femoral fractures (10, 11).

Given Japan's entry into the “100-year life period,” where longevity has become the norm, it is paramount to evaluate the efficacy of surgical intervention in proximal femoral fractures in this unique age group (12, 13). Therefore, the primary objective of this study was to investigate the postoperative life expectancy, changes in walking ability, and alterations in living environments among older adult patients aged ≥100 years who have experienced proximal femoral fractures. The second objective was to determine the safety and efficacy of early surgery within 48 h in centenarians.

## 2 Materials and methods

### 2.1 Ethics approval

All procedures involving human participants in this study adhered to the principles outlined in the Declaration of Helsinki and were approved by the Research Ethics Committee of Shingu Municipal Medical Center (approval number: 94). Written informed consent was obtained from all participants.

### 2.2 Study population

**Figure 1:**
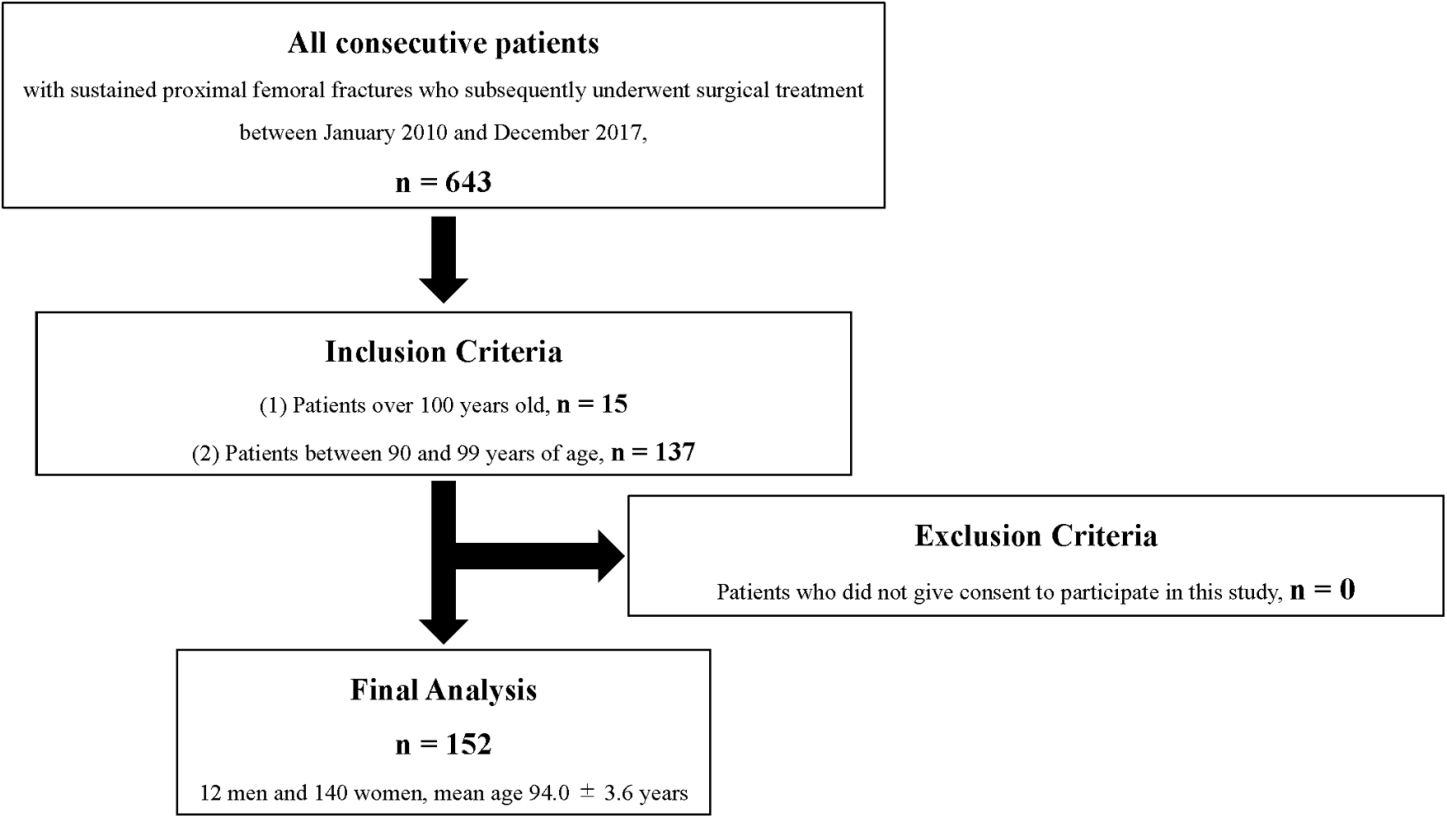
Flow diagram of the present study.

Between January 2010 and December 2017, a total of 643 consecutive patients who experienced sustained proximal femoral fractures and subsequently underwent surgical treatment were enrolled in this study. First, to investigate the postoperative life expectancy, changes in walking ability, and changes in living environment, all patients aged ≥100 years who underwent surgical treatment of proximal femoral fractures within the abovementioned timeframe were included in this study. Next, to elucidate the safety and efficacy of early surgery within 48 h in patients ≥100 years, the patients in their 90s who underwent surgical treatment of proximal femoral fractures between January 2010 and December 2017 were included in the control group. As a result, this retrospective cohort study included 15 older patients aged ≥100 years with proximal femoral fractures who underwent surgery between 2010 and 2017 and 137 patients in their 90s who underwent surgery during the same period who served as the control group (Figure 1).

### 2.3 Evaluation items

The patient background factors included sex; age; body mass index (BMI); American Society of Anesthesiologists (ASA) score (14); presence of dementia; and history of anticoagulant and antiplatelet medication use. We also assessed the fracture type, waiting time from the initial visit to surgery, surgical procedure, operative time, blood loss, and transfusion volume.

The following outcome measures were assessed to evaluate the effectiveness of the surgical treatment in patients with proximal femoral fractures.

1. Rates of perioperative complications: Any complications related to surgery or immediate postoperative period (15).
2. Length of hospital stay: The duration of hospitalization from admission to discharge (16).
3. Proportion of patients discharged to the same facility or home as before the fracture: The percentage of patients who could return to their previous facility or home environment (17).
4. Rate of regaining walking ability: The proportion of patients who regained their ability to walk independently or with minimal assistance (18). The data of patients who were unable to walk before surgery were excluded from the analysis. The information on the ability to walk before the fracture was obtained from the patient or a family member. Moreover, the ability to walk 30 days after surgery was determined by a physician.
5. One-year survival rate: The percentage of patients who survived for 1 year after surgery (19).

### 2.4 Statistical analysis

First, to evaluate the safety and efficacy of surgical treatment in older patients ≥100 years of age with proximal femoral fractures, a comparison was made with the control group of patients in their 90s. Second, to evaluate the effectiveness of early surgery, we compared the preoperative background and outcome measures among three groups: individuals who underwent early surgery at the age of ≥100 years (100s group), those who underwent early surgery in their 90s (90s + early group), and those who did not undergo early surgery in their 90s (90s + late group). To compare the data between the two groups, Fisher's exact or chi-square tests were performed for proportional variables. The Wilcoxon rank-sum test was used to compare continuous variables. An analysis of variance was performed to compare the three groups' preoperative backgrounds and outcome measures. If statistically significant differences were identified, the Bonferroni test was performed to compare the outcomes among the three groups. All statistical analyses were performed using JMP® Pro version 16 (SAS Inc., Cary, NC, USA), and *P*-values < 0.05 were considered statistically significant.

### 2.5 Surgical procedures and perioperative management, rehabilitation

The surgical technique was discussed and decided by five specialists certified by the Japanese Orthopedic Association. As a basic strategy, femoral intramedullary nail and hip screw were selected for all trochanteric fracture cases and neck fracture with minimal dislocation, respectively. Bipolar hip arthroplasty (BHA) was performed for other neck fractures. BHA was standardized to an anterolateral approach and a cementless stem was used. Perioperative pain management comprised regular oral administration of non-steroidal anti-inflammatory drugs and acetaminophen. However, no nerve blocks or other concomitant measures were used. In addition, elastic stockings and foot pumps were uniformly used during the perioperative period as a preventive measure against venous thromboembolism (VTE). Rehabilitation by a physical therapist was started the day after surgery.

## 3 Results

### 3.1 Preoperative comparison between age groups

We initially compared the preoperative characteristics between patients in their 90s and those aged ≥100 years. Notably, age and ASA class demonstrated statistically significant differences, with the 90s group exhibiting a higher prevalence of ASA class III or higher (Table 1). However, no significant differences were observed in other baseline characteristics including sex; BMI; the presence of dementia; history of anticoagulant and antiplatelet medication use; and fracture type.

**Table 1:** Comparison of preoperative characteristics between patients over 100 years of age and those in their 90s.

Characteristics	Patients aged in their 90s	Patients aged over 100 years	*P*-value
Patients (male/female)	137 (10/127)	15 (2/13)	0.411
Age (years)	93.1 ± 2.7	101.4 ± 1.8	<0.001*
BMI (kg/m^2^)	19.3 ± 3.4	19.9 ± 1.1	0.331
ASA class (Ⅰ/Ⅱ/Ⅲ/Ⅳ or more)	17/105/15/0	3/11/0/1	0.010*
Presence of dementia (%)	69.3 (95/137)	66.7 (10/15)	0.831
History of anticoagulant or antiplatelet medication use (%)	21.2 (29/137)	6.7 (1/15)	0.180
Type of fracture (neck/trochanteric)	49/88	6/9	0.746

BMI, body mass index; ASA class, American Society of Anesthesiologists; neck, femoral neck fracture; trochanteric, femoral trochanteric fracture.

* *P* < 0.05 was considered statistically significant. Data are presented as mean ± standard deviation (range) or number.

### 3.2 Postoperative analysis between age groups

Following surgical intervention, we performed a comprehensive postoperative analysis to assess the various outcomes and complications between the two age groups. Notably, no statistically significant differences were identified in the surgical procedure, operative time, blood loss, transfusion volume, perioperative complication rate, hospital stay, percentage of patients who returned to their original living environment, rate of regaining walking ability, and 1-year survival rate (Table 2). However, a notable discrepancy was observed in the waiting time from the initial visit to surgery, with the 90s group experiencing significantly longer waiting times. No fatal complications due to VTE were observed in either group.

**Table 2:** Comparison of postoperative outcomes between patients over 100 years of age and those in their 90s.

Postoperative outcome	Patients aged in their 90s	Patients aged over 100 years	*P*-value
Waiting time (hours)	91.5 ± 65.3	35.2 ± 22.7	0.001*
Surgical procedure (ORIF/BHA)	99/38	9/6	0.320
Operative time (minutes)	55.4 ± 19.5	54.1 ± 18.7	0.816
Blood loss (ml)	121.5 ± 117.3	119.4 ± 97.8	0.896
Blood transfusion volume (ml)	460.7 ± 312.2	480.0 ± 374.0	0.761
Perioperative complications (%)	19.0 (26/137)	13.3 (2/15)	0.592
Hospital stay (days)	42.6 ± 20.4	38.3 ± 21.8	0.449
Returned to original living environment (%)	56.2 (77/137)	80 (12/15)	0.076
Regained walking ability (%)	51.4 (55/107)	60 (6/10)	0.603
One-year survival rate (%)	89.8 (123/137)	93.3 (14/15)	0.661

Waiting time, waiting time from visit to surgery; ORIF, open reduction and internal fixation; BHA, bipolar hip arthroplasty; Returned to original living environment, the percentage of patients who were able to return to their previous facility or home environment; Regained walking ability, the proportion of patients who regained the ability to walk independently or with minimal assistance. Data of patients who did not have the ability to walk before surgery were excluded from the calculation.

* *P* < 0.05 was considered statistically significant. Data are presented as mean ± standard deviation (range) or number.

### 3.3 Factors influencing early surgery in the 90s group

A significant number of patients in the 90s group did not undergo early surgery within 48 h, primarily due to non-patient factors (approximately 60%), followed by a history of anticoagulant or antiplatelet medication use (approximately 31%) and comorbidity limitations (approximately 29%). Among the non-patient factors, a substantial proportion of delays were attributed to the injury having been sustained during the national holiday period (approximately 60%).

### 3.4 Comparative assessment of early surgery efficacy

Further analysis was performed to evaluate the effectiveness of early surgery by comparing the outcomes among the three groups:

1. Patients who underwent early surgery at an age ≥100 years
2. Patients who underwent early surgery in their 90s
3. Patients who did not undergo early surgery in their 90s

Distinct differences between these groups were observed in the ASA class and history of anticoagulant and antiplatelet medication use (Table 3).

**Table 3:** Comparison of preoperative characteristics between patients who did and did not undergo early surgery.

Characteristics	Early surgery patients in their 90s	Late surgery patients in their 90s	Early surgery patients ≥100 years	*P*-value
Patients (male/female)	54 (5/49)	83 (5/78)	15 (2/13)	0.563
Age (years)	93.8 ± 2.6	92.7 ± 2.7	101.4 ± 1.8	<0.001*
BMI (kg/m^2^)	19.2 ± 2.7	19.3 ± 3.9	19.9 ± 1.1	0.618
ASA class (Ⅰ/Ⅱ/Ⅲ/Ⅳ or more)	9/45/0/0	8/60/15/0	3/11/0/1	0.001*
Presence of dementia (%)	57.4 (31/54)	77.1 (64/83)	66.7 (10/15)	0.051
History of anticoagulant or antiplatelet medication use (%)	5.6 (3/54)	31.3 (26/83)	6.7 (1/15)	<0.001*
Type of fracture (neck/trochanteric)	18/36	31/52	6/9	0.846

BMI, body mass index; ASA class, American Society of Anesthesiologists; neck, femoral neck fracture; trochanteric, femoral trochanteric fracture.

* *P* < 0.05 was considered statistically significant. Data are presented as mean ± standard deviation (range) or number.

### 3.5 Postoperative outcomes among the three groups

**Figure 2:**
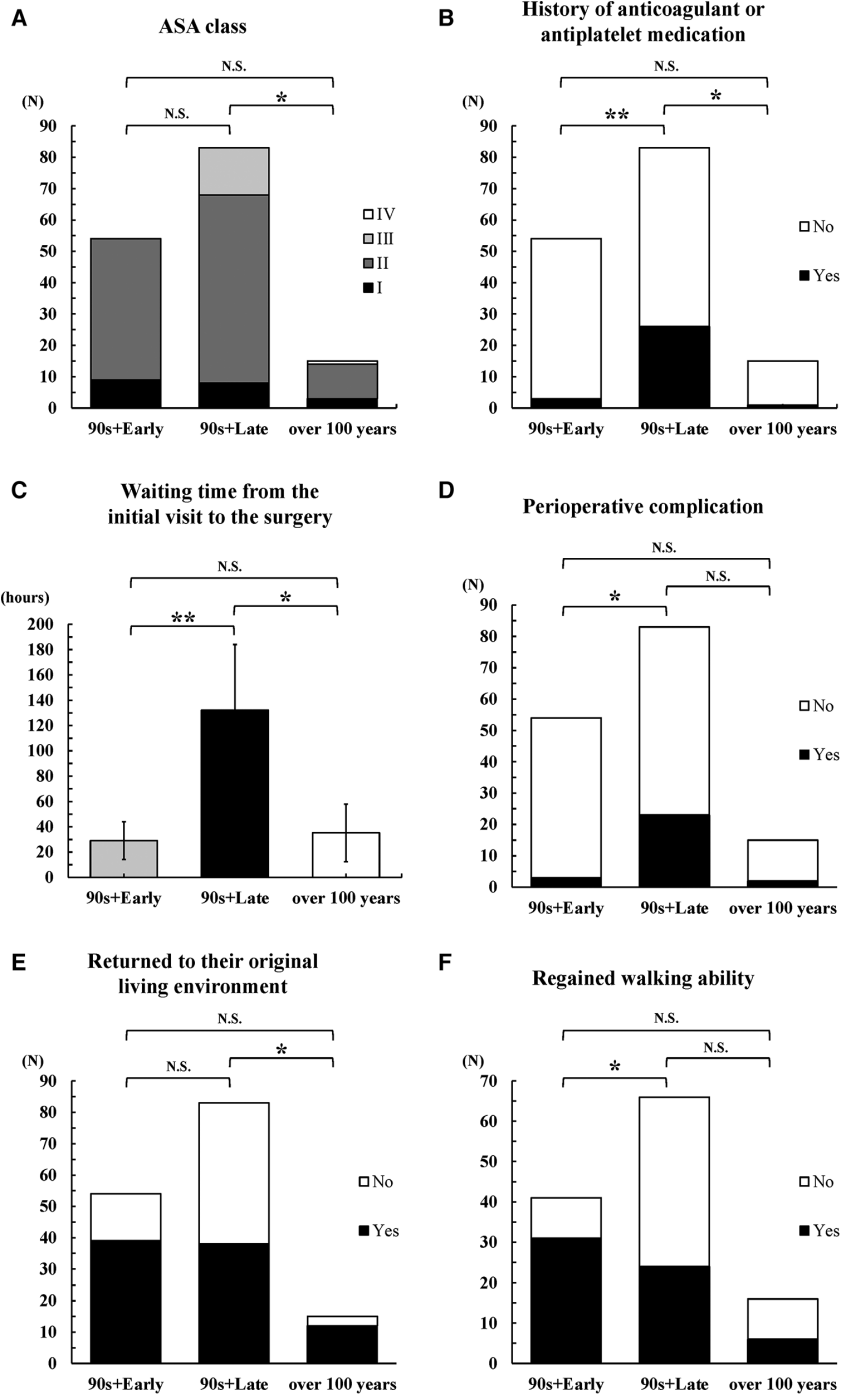
Postoperative outcomes among the three groups. 90s + early: Patients who underwent early surgery in their 90s; 90s + late: Patients who did not undergo early surgery in their 90s; over 100 years: Patients who underwent early surgery at ≥100 years of age. (**A**) ASA class, (**B**) history of anticoagulant and antiplatelet medications, (**C**) waiting time from the initial visit to surgery, (**D**) perioperative complications, (**E**) patients who returned to their original living environment, (**F**) patients who regained their walking ability. **P < 0.0167, *0.0167 ≤ *P* < 0.05, N.S: *P* ≥ 0.05.

Postoperative outcomes demonstrated notable differences in the rate of perioperative complications, percentage of patients who returned to their original living environment, and rate of regaining walking ability (Table 4). Although statistical significance was not consistently observed, the trends suggested that the 90s + late group tended to have more perioperative complications and poorer ambulatory ability than did the 90s + early group (Figure 2). Moreover, the safety and efficacy of early surgery in the 100s group were similar to those in the 90s + early group.

**Table 4:** Comparison of postoperative outcomes between patients who did and did not undergo early surgery.

	Early surgery patients in their 90s	Late surgery patients in their 90s	Early surgery patients ≥100 years	*P*-value
Waiting time (hours)	29.0 ± 14.9	132.1 ± 51.9	35.2 ± 22.7	<0.001*
Surgical procedure (ORIF/BHA)	39/15	60/23	9/6	0.610
Operative time (minutes)	55.9 ± 19.6	55.7 ± 19.5	54.1 ± 18.7	0.988
Blood loss (ml)	109.2 ± 96.1	129.5 ± 129.2	119.4 ± 97.8	0.676
Blood transfusion volume (ml)	408.9 ± 258.0	494.5 ± 340.1	480.0 ± 374.0	0.448
Perioperative complications (%)	5.6 (3/54)	27.7 (23/83)	13.3 (2/15)	0.004*
Hospital stay (days)	39.6 ± 23.7	44.5 ± 17.8	38.3 ± 21.8	0.086
Returned to original living environment (%)	72.2 (39/54)	45.8 (38/83)	80 (12/15)	0.002*
Regained walking ability (%)	75.6 (31/41)	36.4 (24/66)	60 (6/10)	<0.001*
One-year survival rate (%)	96.3 (52/54)	85.5 (71/83)	93.3 (14/15)	0.108

Waiting time, waiting time from visit to surgery; ORIF, open reduction and internal fixation; BHA, bipolar hip arthroplasty; Returned to their original living environment, the percentage of patients who were able to return to their previous facility or home environment; Regained walking ability, the proportion of patients who regained the ability to walk independently or with minimal assistance. The data of patients who did not have the ability to walk before surgery were excluded from the calculation.

* *P* < 0.05 was considered statistically significant. Data are presented as mean ± standard deviation (range) or number.

These findings collectively underscore the comparability of surgical outcomes between older patients aged ≥100 years and those in their 90s, along with the potential benefits of early surgery for enhancing patient outcomes and minimizing complications within these specific age cohorts.

## 4 Discussion

The abovementioned findings provide valuable insights into the safety and efficacy of surgical treatment in older patients aged ≥100 years with proximal femoral fractures and the impact of early surgery on patient outcomes. Notably, our analysis compared the outcomes of patients ≥100 years of age with those of patients in their 90s, clarifying the daunting challenges and considerations a super-aged society faces. Furthermore, our study thoroughly examined the influence of early surgical intervention on outcomes among various age groups, highlighting the significance of timely intervention in optimizing outcomes. This study provides novel insights by addressing a notable gap in the literature into managing older adult patients aged ≥100 years with proximal femoral fractures. Our examination of the specific impact of surgical treatment on the postoperative life expectancy, changes in walking ability, and alterations in the living environment within this unique age group could provide the limited evidence available in this context. Furthermore, the investigation of the safety and efficacy of early surgery within 48 h in patients ≥100 years of age fills a critical void in understanding fracture management in this demographic. The comprehensive evaluation of multiple outcomes, ranging between perioperative complications and long-term survival, enhanced the robustness and applicability of our findings.

When contextualized within the existing literature, our results align with a broader understanding of surgical interventions for proximal femoral fractures in older adults (6, 7, 20). Although several studies have emphasized the importance of comprehensive assessment, appropriate surgical techniques, and postoperative care in optimizing outcomes (6, 8), few have explicitly addressed the unique needs of older adult patients aged ≥100 years with these fractures (3, 10). This study bridges this gap and extends the evidence base to encompass this distinct demographic group, reinforcing the relevance and timeliness of the findings.

The observed similarities in surgical outcomes between patients aged ≥100 years and those in their 90s highlight the potential for successful management of proximal femoral fractures across these age groups. Our study suggests that age alone may not be the sole determinant of surgical success, as factors such as early surgical intervention and careful preoperative assessment could play pivotal roles in enhancing patient outcomes (21). These findings further support the effectiveness of early surgery within 48 h, showing trends toward improved perioperative complications, ambulatory ability, and return to the original living environment. These insights underscore the importance of prompt surgical intervention in both age cohorts, resonating with the broader understanding of the benefits of early intervention (4, 5, 10, 11, 22, 23).

Although this study offers valuable contributions, it is not exempt from limitations. The retrospective design and single-center setting may have introduced inherent biases and limited the generalizability of our findings. Moreover, our sample size was modest, particularly in the over-100-year age group, warranting cautious interpretation. The fact that all of the centenarians were able to undergo surgery within 48 h may have been a function of the special awareness of their physicians and medical staff. In other words, the numerical specialness of “over 100 years old” may have increased the awareness of the medical staff in charge of prioritizing full-body examinations and emergency surgical responses. We considered this to be one of the limitations of this study as a selection bias. The absence of randomization in our study design may have led to potential confounding, as unmeasured variables could have influenced the observed associations. Although we adjusted for confounding factors in our analyses, unmeasured confounders remain a limitation that could affect the validity of our conclusions. In addition, focusing on the 1-year postoperative survival rate offers a valuable snapshot of short-term outcomes. However, the absence of a long-term follow-up limits our ability to capture potential changes in outcomes beyond the initial recovery period, providing a more comprehensive understanding of the sustainability of surgical benefits in this population. Given the single-center nature of our study, caution is required when extrapolating our findings to other healthcare settings or geographic regions, as variations in healthcare practices, patient demographics, and socioeconomic factors could affect the applicability of our results beyond our study population. We consider it a major limitation that this study focused on actual age and did not consider physiological age such as frailty. This is one of the issues necessitating the need for future research. Despite these limitations, our study could provide valuable preliminary evidence, paving the way for future research with larger multicenter cohorts to validate our results and provide more nuanced insights.

In conclusion, our study elucidated the efficacy of surgical treatment of proximal femoral fractures in older patients aged ≥100 years. We underscored the comparability of surgical outcomes across various age groups and highlighted the potential benefits of surgical intervention within 48 h. The findings of this study have important implications for clinicians, policymakers, and patients. Clinicians are encouraged to consider early surgical intervention as a viable approach for improving outcomes in older patients with proximal femoral fractures. Policymakers can use our results to shape healthcare strategies tailored to the continuing challenges of a super-aged society, emphasizing the need for targeted fracture management approaches. Ultimately, patients and their families could draw confidence from evidence-based insights that guide decision-making and foster informed discussions with healthcare providers. Although further research is warranted to validate and expand our findings, this study provides a foundational understanding of optimal fracture management in a rapidly aging population.

## Data Availability

The original contributions presented in the study are included in the article/Supplementary Material, further inquiries can be directed to the corresponding author.

## References

[1] Cooper C Campion G Melton LJ. Hip fractures in the elderly: a world-wide projection. Osteoporos Int. (1992) 2:285–9. 10.1007/BF01623184 1421796

[2] Health Mo Welfare La. Annual Report of Long-Term Care Insurance. Tokyo, Japan: Ministry of Health, Labour and Welfare (2021).

[3] Paksima N Koval KJ Aharanoff G Walsh M Kubiak EN Zuckerman JD Predictors of mortality after hip fracture: a 10-year prospective study. Bull NYU Hosp Jt Dis. (2008) 66:111–7. 18537780

[4] Orosz GM Magaziner J Hannan EL Morrison RS Koval K Gilbert M Association of timing of surgery for hip fracture and patient outcomes. JAMA. (2004) 291:1738–43. 10.1001/jama.291.14.1738 15082701 PMC1454713

[5] Simunovic N Devereaux PJ Sprague S Guyatt GH Schemitsch E Debeer J Effect of early surgery after hip fracture on mortality and complications: systematic review and meta-analysis. CMAJ. (2010) 182:1609–16. 10.1503/cmaj.092220 20837683 PMC2952007

[6] Bhandari M Swiontkowski M. Management of acute hip fracture. N Engl J Med. (2017) 377:2053–62. 10.1056/NEJMcp1611090 29166235

[7] Maffulli N Aicale R. Proximal femoral fractures in the elderly: a few things to know, and some to forget. Medicina (Kaunas). (2022) 58:1314. 10.3390/medicina58101314 36295475 PMC9612001

[8] Magaziner J Hawkes W Hebel JR Zimmerman SI Fox KM Dolan M Recovery from hip fracture in eight areas of function. J Gerontol A Biol Sci Med Sci. (2000) 55:M498–507. 10.1093/gerona/55.9.m498 10995047

[9] Hu F Jiang C Shen J Tang P Wang Y. Preoperative predictors for mortality following hip fracture surgery: a systematic review and meta-analysis. Injury. (2012) 43:676–85. 10.1016/j.injury.2011.05.017 21683355

[10] Shiga T Wajima Z Ohe Y. Is operative delay associated with increased mortality of hip fracture patients? Systematic review, meta-analysis, and meta-regression. Can J Anaesth. (2008) 55:146–54. 10.1007/BF03016088 18310624

[11] Vidán MT Sánchez E Gracia Y Marañón E Vaquero J Serra JA. Causes and effects of surgical delay in patients with hip fracture: a cohort study. Ann Intern Med. (2011) 155:226–33. 10.7326/0003-4819-155-4-201108160-00006 21844548

[12] Oliver D Griffiths R Roche J Sahota O. Hip fracture. BMJ Clin Evid. (2010) 2010:1110. 21726483 PMC2907602

[13] Neuman MD Silber JH Magaziner JS Passarella MA Mehta S Werner RM. Survival and functional outcomes after hip fracture among nursing home residents. JAMA Intern Med. (2014) 174:1273–80. 10.1001/jamainternmed.2014.2362 25055155 PMC4122620

[14] Owens WD Felts JA Spitznagel EL Jr. ASA physical status classifications: a study of consistency of ratings. Anesthesiology. (1978) 49:239–43. 10.1097/00000542-197810000-00003 697077

[15] Dix DB Araoye IB Staggers JR Lin CP Shah AB Agarwal AK A systematic review and meta-analysis of complications in conversion arthroplasty methods for failed intertrochanteric fracture fixation. J Clin Orthop Trauma. (2019) 10:282–5. 10.1016/j.jcot.2018.02.007 30828194 PMC6383065

[16] Tayyebi H Hasanikhah M Heidarikhoo M Fakoor S Aminian A. Length of hospital stay and mortality of hip fracture surgery in patients with coronavirus disease 2019 (COVID-19) infection: a systematic review and meta-analysis. Curr Orthop Pract. (2022) 33:172–7. 10.1097/BCO.0000000000001087 35222789 PMC8862679

[17] Ahmed HE Zourob E Lukic J Latimer L Anto J Rajeev A. Proximal femoral fracture outcomes in inpatients and community patients: a comparative study. J Frailty Sarcopenia Falls. (2021) 6:218–24. 10.22540/JFSF-06-218 34950812 PMC8649859

[18] Carneiro MB Alves DP Mercadante MT. Physical therapy in the postoperative of proximal femur fracture in elderly. Literature review. Acta Ortop Bras. (2013) 21:175–8. 10.1590/S1413-78522013000300010 24453665 PMC3861999

[19] Merchán-Galvis AM Muñoz-García DA Solano F Velásquez JC Sotelo NF Molina DA Delayed surgery and health related quality of life in patients with proximal femoral fracture. Sci Rep. (2023) 13:11131. 10.1038/s41598-023-33592-3 37429947 PMC10333196

[20] Vidán M Serra JA Moreno C Riquelme G Ortiz J. Efficacy of a comprehensive geriatric intervention in older patients hospitalized for hip fracture: a randomized, controlled trial. J Am Geriatr Soc. (2005) 53:1476–82. 10.1111/j.1532-5415.2005.53466.x 16137275

[21] Wall PV Mitchell BC Ta CN Kent WT. Review of perioperative outcomes and management of hip fracture patients on direct oral anticoagulants. EFORT Open Rev. (2023) 8:561–71. 10.1530/EOR-22-0060 37395711 PMC10321051

[22] Espinosa KA Gélvez AG Torres LP García MF Peña OR. Pre-operative factors associated with increased mortality in elderly patients with a hip fracture: a cohort study in a developing country. Injury. (2018) 49:1162–8. 10.1016/j.injury.2018.04.007 29674111

[23] Fischer H Maleitzke T Eder C Ahmad S Stöckle U Braun KF. Management of proximal femur fractures in the elderly: current concepts and treatment options. Eur J Med Res. (2021) 26:86. 10.1186/s40001-021-00556-0 34348796 PMC8335457

